# Interleukin 6, lipopolysaccharide-binding protein and interleukin 10 in the prediction of risk and etiologic patterns in patients with community-acquired pneumonia: results from the German competence network CAPNETZ

**DOI:** 10.1186/1471-2466-12-6

**Published:** 2012-02-20

**Authors:** Katrin Zobel, Peter Martus, Mathias W Pletz, Santiago Ewig, Michael Prediger, Tobias Welte, Frank Bühling

**Affiliations:** 1 Department of Pneumology, Carl-Thiem-Klinikum Cottbus, Thiemstrasse 111, 03048 Cottbus, Germany; 2 Department of Biometry and Clinical Epidemiologie, Charité, Chariteplatz 1, 10098 Berlin, Germany; 3 Division of Gastroenterology, Hepatology and Infectious Diseases, Jena University Hospital, Hospital Erlanger Allee 101, 07740 Jena, Germany; 4 Thoraxzentrum Ruhrgebiet, Respiratory and Infectious Diseases, Ev. Krankenhaus Herne und Augusta-Kranken-Anstalt, Bochum, Thoraxzentrum Ruhrgebiet, Hordeler Strasse 7-9, 44651 Herne, Germany; 5 Department of Pneumology, Hannover Medical School, Carl-Neuberg-Strasse 1, 30625 Hannover, Germany; 6 Department of Laboratory Medicine, Carl-Thiem-Klinikum Cottbus, Thiemstrasse 111, 03048 Cottbus, Germany

**Keywords:** Interleukin-6, Interleukin-10, LBP, CAP, Pneumonia, Biomarker, Cytokine

## Abstract

**Background:**

The aim of our study was to investigate the predictive value of the biomarkers interleukin 6 (IL-6), interleukin 10 (IL-10) and lipopolysaccharide-binding protein (LBP) compared with clinical CRB and CRB-65 severity scores in patients with community-acquired pneumonia (CAP).

**Methods:**

Samples and data were obtained from patients enrolled into the German CAPNETZ study group. Samples (blood, sputum and urine) were collected within 24 h of first presentation and inclusion in the CAPNETZ study, and CRB and CRB-65 scores were determined for all patients at the time of enrollment. The combined end point representative of a severe course of CAP was defined as mechanical ventilation, intensive care unit treatment and/or death within 30 days. Overall, a total of 1,000 patients were enrolled in the study. A severe course of CAP was observed in 105 (10.5%) patients.

**Results:**

The highest IL-6, IL-10 and LBP concentrations were found in patients with CRB-65 scores of 3-4 or CRB scores of 2-3. IL-6 and LBP levels on enrollment in the study were significantly higher for patients with a severe course of CAP than for those who did not have severe CAP. In receiver operating characteristic analyses, the area under the curve values for of IL-6 (0.689), IL-10 (0.665) and LPB (0.624) in a severe course of CAP were lower than that of CRB-65 (0.764) and similar to that of CRB (0.69). The accuracy of both CRB and CRB-65 was increased significantly by including IL-6 measurements. In addition, higher cytokine concentrations were found in patients with typical bacterial infections compared with patients with atypical or viral infections and those with infection of unknown etiology. LBP showed the highest discriminatory power with respect to the etiology of infection.

**Conclusions:**

IL-6, IL-10 and LBP concentrations were increased in patients with a CRB-65 score of 3-4 and a severe course of CAP. The concentrations of IL-6 and IL-10 reflected the severity of disease in patients with CAP. The predictive power of IL-6, IL-10 and LBP for a severe course of pneumonia was lower than that of CRB-65. Typical bacterial pathogens induced the highest LBP, IL-6 and IL-10 concentrations.

## Background

Community-acquired pneumonia (CAP) is the most common potentially fatal infectious disease in industrialized countries [1,2]. In order to classify pneumonia severity and to guide treatment decisions, several prognostic scores for CAP have been developed. The CURB-65 and CRB-65 scores are currently the preferred scores in Europe because of their simplicity and applicability. The CRB score has been shown to be less useful for the estimation of the risk of death within 30 days [3,4].

A variety of pro- and anti-inflammatory cytokines are involved in the regulation of the inflammatory response to pulmonary infections; measurements of these cytokines have been used to improve diagnosis and assess severity, and thus predict the prognosis of CAP. The concentrations of interleukin 6 (IL-6) [5] and interleukin 10 (IL-10) [6] reflect inflammatory and anti-inflammatory reactions, respectively. The concentration of lipopolysaccharide-binding protein (LBP) is increased during bacterial infections [7].

IL-6 is secreted by a variety of cells such as T-lymphocytes, macrophages, endothelial cells and epithelial cells. In inflammatory reactions the rise of IL-6 concentration precedes that of most of the other cytokines [5,7-9] and it has a plasma half-life of 20-60 min. IL-10 is an anti-inflammatory cytokine that is produced primarily by monocytes and to a lesser extent by Th-2 lymphocytes. IL-10 inhibits the synthesis of pro-inflammatory cytokines and suppresses antigen presentation, and normal serum half-life is only a few hours [6]. LBP is an acute-phase protein that is increased 30-fold within 24-48 h of infection [7] and it forms complexes with bacterial lipopolysaccharides. These complexes bind to specific receptors on monocytes and macrophages and boost inflammation. The serum half-life of LBP is 2-4 h.

The aim of our study was to investigate the predictive value of IL-6, IL-10 and LBP compared with the CRB-65 score with respect to the clinical course of CAP and the microbial etiology.

## Methods

### Study population

CAPNETZ is a German competence network for the study of CAP. It collects data and samples (blood, urine and sputum) from patients with proven CAP (hospitalized patients and outpatients). The network consists of local clinical centers throughout Germany [1].

Samples and patient data were obtained from CAPNETZ study group. All cases of CAP were reported, via a network of sentinel practices and hospitals, to the study monitor of the corresponding local clinical center.

The recruitment period for the present study was January 2001 to December 2006. A total of 3,220 subjects were selected from the database. The population included 105 subjects with a severe course of CAP, which was defined as mechanical ventilation, intensive care unit (ICU) treatment and/or death within 30 days of involvement. These 105 subjects, and a random sample of 895 controls stratified for CRB-65 score, were included in the study. The control group was defined using the algorithm described as follows. First, the total number of evaluable cases was determined, *n *= 105. The total size of the study population was chosen in order to get 1,000 probes for analysis. Thus with 105 cases, 895 controls had to be selected. For the selection of the control sample population, the number of subjects with CRB-65 0-3 was fixed to match the frequency distribution of CRB-65 in all controls. Then, the respective controls were selected from all controls in each CRB-65 stratum using the random number generator of SPSS software (SPSS, Chicago, Illinois, USA).

Samples (blood, sputum and urine) were taken within 24 h of first presentation and inclusion in the CAPNETZ study.

The inclusion criteria were: age ≥ 18 years, a new pulmonary infiltrate diagnosed by chest radiograph, written informed consent prior to inclusion in the study, and at least one of the following clinical symptoms: cough with purulent sputum, fever or positive auscultation.

The exclusion criteria were: acquired or therapeutically induced immune deficiency, active tuberculosis, or hospitalization less than 4 weeks prior to infection.

The decision where (inpatient or outpatient) and how to treat the patient was made by the attending physician.

All patients were assessed at first presentation and during follow-up according to a standardized web-based data sheet. After 14, 28 and 180 days, all patients were contacted either in person or via telephone for a structured interview on outcome parameters (for example: resolution of symptoms, length of antimicrobial therapy or death) [10,11].

The CAPNETZ project was approved by the local ethics committee. Written informed consent was obtained from each patient prior to his/her inclusion in the study.

### Determination of CRB and CRB-65 scores

For all patients the CRB and CRB-65 scores were determined on inclusion in the study [10]. The CRB score consists of three variables: confusion, respiratory rate ≥ 30/min, and systolic blood pressure < 90 mmHg or diastolic blood pressure ≤60 mmHg. The CRB-65 score adds age ≥ 65 years to the CRB score, and one point is given for each parameter present. CRB scores range from 0 to 3, and CRB-65 scores range from 0 to 4.

### Measurement of IL-6, LBP and IL-10

All serum samples to be tested for IL-6, LBP and IL-10 were stored at -70°C. The cytokine concentrations were measured using a solid-phase, two-site chemiluminescence immunometric assay on Immulite 1000 (LBP and IL-10) or Immulite 2000 (IL-6; Siemens Healthcare, Eschborn, Germany) according to the manufacturer's instructions. The detection limits are 1.2 μg/ml, 0.99 pg/ml and 2 pg/ml for LBP, IL-10 and IL-6, respectively.

### Microbiologic diagnosis

Potential pathogens were detected using microbiologic, serologic and molecular biological standard procedures, as described previously [11,12]. *Streptococcus pneumoniae, Haemophilus influenzae, Klebsiella pneumoniae, Escherichia coli *and other enterobacterial species, *Pseudomonas aeruginosa, Moraxella catarrhalis *and *Stenotrophomonas maltophilia *were regarded as definite causative pathogens when they were isolated from: (1) blood cultures or pleural fluid cultures, or (2) good-quality sputum containing < 25 polymorphonuclear cells and < 10 epithelial cells per high-power field (total magnification × 1,000), and there was predominant growth from culture of the sputum of > 10^6 ^colony-forming units (CFU)/ml or > 10^4 ^CFU/ml from bronchoalveolar lavage fluid. If one of these criteria was not met, the presence of any of the bacteria listed above was judged to be indeterminate. In addition, *Chlamydia pneumoniae *(IgM positive ≥ 1:32 and/or positive PCR), *Legionella *spp. (culture, positive urinary antigen and/or PCR), *Mycoplasma pneumoniae *(PCR), *S. pneumoniae *(urinary antigen), influenza A and B viruses, respiratory syncytial virus, adenovirus, enterovirus (all PCR) and *Aspergillus *spp. (only with concomitant lung abscess and/or histologic confirmation) were considered to be causative pathogens.

### Statistics

Statistical analyses were performed with SPSS software. Two-group comparisons of non-parametric data were calculated by using a *t*-test. For multigroup comparisons, one-way ANOVA with Bonferroni post-hoc analyses were used. No correction for multiple testing was applied with respect to the three cytokines (IL-6, IL-10 and LBP). The operating characteristics of IL-6, IL-10 and LBP were assessed by calculating sensitivity, specificity and predictive values.

We constructed receiver-operating characteristic (ROC) curves and determined the area under the curve (AUC). The combination of parameters was done using the linear predictor obtained from logistic regression analysis. Thus, no categorization was applied. The usefulness of the markers for improving the prediction by CRB-65 score was evaluated by calculation of continuous net reclassification improvement (NRI) as described by Pencina *et al. *[13,14]. Thus, we looked at the rates of increased and decreased event probability for each subject without applying a categorization.

*P *values below 0.05 (two-sided) were considered to be statistically significant. A comparison of ROC curves was done by using the method of DeLong [15], which takes into account the correlation of measurements and increases the power considerably, compared with the 'naïve' method, which uses raw standard errors.

Samples from patients who did not have a severe course of CAP were used for all controls, but the percentage of patients with a severe course of CAP was about threefold (10.5% versus 3.3%) more in the final study population as compared with that in the primary sample of 3,220 subjects. Thus, sensitivity and specificity of scores could be determined without further correction; however, predictive values had to be corrected using the formula of Bayes.

## Results

### Patients

#### Epidemiology

Overall, a total of 1,000 patients with a mean age of 58.7 ± 18 years (range 18-95 years) were enrolled in the study. Of these, 565 patients were men and 435 were women. A total of 596 patients were hospitalized and 404 patients were treated as outpatients. One hundred and five patients (75 men, 30 women) were found to have a severe course of CAP, defined as mechanical ventilation, ICU treatment, and/or death within 30 days of involvement. Forty-seven of the 49 ICU patients needed mechanical ventilation and eight patients died within 30 days. Fifty-six patients who were not treated in the ICU died within 30 days.

#### Distribution of CRB-65 scores

The numbers of patients with different CRB and CRB-65 risk scores are given in Table 1.

**Table 1:** Numbers of patients with defined risk scores for CRB and CRB-65, and the corresponding number of patients with a severe course of CAP defined as mechanical ventilation, intensive care unit (ICU) treatment and/or death within 30 days of involvement

Risk score	CRB		CRB-65	
	**Total no**.	No. with severe course	**Total no**.	No. with severe course
0	727	41	418	11
1	242	50	425	43
2	28	11	135	39
3-4	3	3	22	12

We identified 277 patients who had received antibiotic pretreatment within 28 days before enrollment in the study. The CRB-65 score was significantly lower in patients who had antibiotic pretreatment (0.64 ± 0.717) compared with those who did not have pretreatment (0.81 ± 0.782, *P *< 0.002).

The concentrations of LBP and IL-10 were significantly lower in patients who had antibiotic pretreatment (18.78 ± 13.3 μg/ml and 4.35 ± 5.69 pg/ml, respectively) compared with patients who did not (22.81 ± 16.73 μg/ml, *P *< 0.001, and 6.98 ± 18.73 pg/ml, *P *< 0.022). The difference in IL-6 concentration was close to significance in patients who had pretreatment (72.19 ± 332.71 pg/ml) compared with those who had not had pretreatment (202.91 ± 1, 102.57 pg/ml, *P *= 0.053).

### Cytokines in severe pneumonia

**Figure 1:**
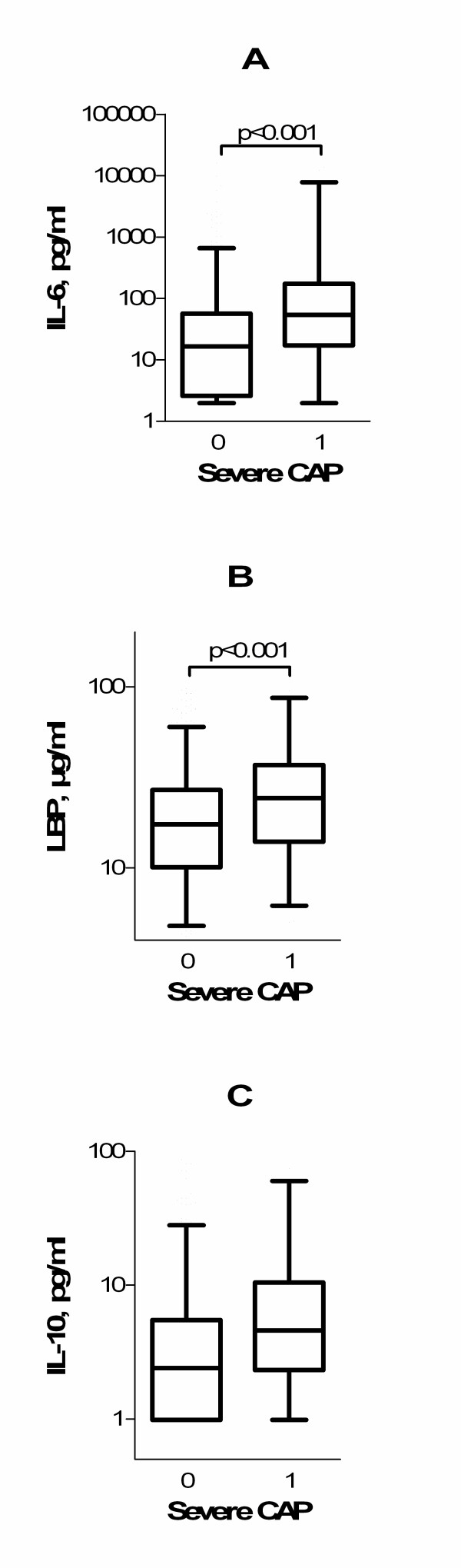
**Levels of cytokines in the course of community-acquired pneumonia**. **(A) **Interleukin 6 (IL-6), **(B) **lipopolysaccharide-binding protein (LBP), and **(C) **interleukin 10 (IL-10). Boxes: horizontal lines indicate 25th to 75th percentiles, and whiskers indicate median, 2.5th and 97.5th percentiles. CAP, community-acquired pneumonia.

Median concentrations of IL-6 and LBP on patient enrollment were significantly higher in patients with a severe course of CAP compared with patients who did not have a severe course of CAP (54.4 pg/ml versus 16.6 pg/ml, *P *< 0.001, and 24.3 μg/ml versus 17.4 μg/ml, *P *< 0.001, respectively; Figure 1A,B). The difference in IL-10 concentrations was close to significance (*P *= 0.053; Figure 1C).

**Figure 2:**
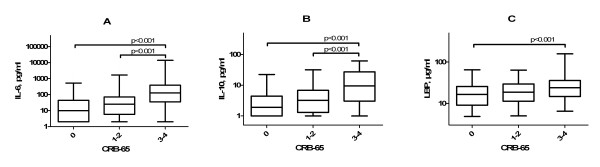
**Levels of cytokines and CRB-65 class on patient admission in community-acquired pneumonia**. **(A) **Interleukin 6 (IL-6), **(B) **lipopolysaccharide-binding protein (LBP), and **(C) **interleukin 10 (IL-10). Continuous lines denote median values, boxes represent 25th to 75th percentiles and whiskers indicate 2.5th and 97.5th percentiles. CAP, community-acquired pneumonia.

High CRB-65 and CRB (data not shown) scores were associated with a severe course of pneumonia. Figure 2 shows that IL-6, IL-10 and LBP levels in patients with CRB-65 score 0 were significantly lower than those in patients with CRB-65 score 3-4 (*P *< 0.001). The respective median values were: 9.92 pg/ml versus 127.5 pg/ml for IL-6, 1.9 pg/ml versus 9.5 pg/ml for IL-10, and 16.45 μg/ml versus 23.95 μg/ml for LBP. There was also a significant difference (*P *< 0.001) between IL-6 and IL-10 concentrations in patients with CRB-65 score 1-2 and those with CRB-65 score 3-4 (median values: 25.1 pg/ml versus 127.5 pg/ml, and 3.2 pg/ml versus 9.5 pg/ml, respectively).

### Predictive potential of cytokines

**Figure 3:**
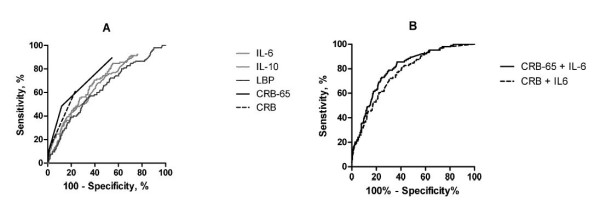
**Receiver-operating characteristic curves to predict a severe course of community-acquired pneumonia and high CRB-65 or CRB scores**. **(A) **Receiver operator characteristic (ROC) curves for interleukin 6 (IL-6; area under curve (AUC) 0.689; 95% CI 0.636 to 0.741; *P *< 0.001), lipopolysaccharide-binding protein (LBP; AUC 0.624; 95% CI 0.567 to 0.681; *P *< 0.0001), interleukin 10 (IL-10; AUC 0.665; CI 0.611 to 0.718; *P *< 0.0001), CRB score (AUC 0.701; 95% CI 0.643 to 0.760; *P *< 0.0001) and CRB-65 score (AUC 0.764; 95% CI 0.716 to 0.812; *P *< 0.0001), and **(B) **combination of CRB-65 score plus IL-6 concentration (AUC 0.800; 95% CI 0.759 to 0.842; *P *< 0.0001) and CRB score plus IL-6 concentration (AUC 0.765; 95% CI 0.720 to 0.810; *P *< 0.0001), in predicting a severe course of community-acquired pneumonia (CAP).

ROC curves illustrate the accuracy of IL-6, LBP and IL-10 concentrations, and CRB-65 and CRB scores, in predicting a severe course of pneumonia (Figure 3A,B). The best discriminatory power was shown by a combination of CRB-65 score and IL-6 concentration (AUC 0.800), and a combination of CRB score and IL-6 (AUC 0.765). The AUC for IL-6 was 0.689, which demonstrated moderate discriminatory power. The AUC values for LBP (0.624) and IL-10 (0.665) were lower. The combined use of IL-6, LBP and IL-10 (AUC 0.689) or IL-6/IL-10 ratio (AUC 0.63) did not significantly improve the accuracy to predict events, compared with IL-6, LBP or IL-10 alone. CRB-65 score alone had a higher discriminatory power, with an AUC of 0.764. The operating characteristics of the CRB score were similar to those of the cytokines (AUC 0.701).

In addition, the accuracy of cytokine concentrations in predicting death within 30 days and mechanical ventilation were analyzed separately with a lower statistical power because of the lower number of events. The best discriminatory power for death within 30 days was shown with IL-6 (AUC 0.646; 95% CI 0.579 to 0.714; *P *< 0.0001). CRB-65 score alone had a lower discriminatory power, with an AUC of 0.626 (95% CI 0.548 to 0.705; *P *< 0.001). The AUC values for IL-10 (0.574) and LBP (0.567) were not significantly different from 0.50. IL-10 had a better discriminatory power for mechanical ventilation (AUC 0.710; 95% CI 0.598 to 0.813; *P *< 0.0001) compared with IL-6 (AUC 0.644; 95% CI 0.503 to 0.784; *P *< 0.0001). CRB-65 score alone had a higher discriminatory power for this mechanical ventilation, with an AUC of 0.777 (95% CI 0.669 to 0.884; *P *< 0.001). The AUC for LBP (0.612) was not significant.

Based on the finding that a combination of IL-6 concentration and CRB-65 score had the best discriminatory power, we calculated whether the addition of IL-6 concentration to CRB-65 score improved the classification of individual patients by calculating the NRI. Using IL-6 in addition to CRB-65, we obtained an NRI of 0.21 (CI 0.02 to 0.40) for patients who reached the combined end point representative of a severe course of CAP and an NRI of 0.15 (CI 0.09 to 0.21) for the control group. The NRI for the complete population (a combination of the NRI of the controls and the patients with the combined end point) was increased significantly (0.36, CI 0.16 to 0.56). The addition of IL-6 led to an up classification of 60.6% of patients with a severe course of CAP and a down classification of 57.4% of control patients (Table 2) and thus improved the categorization.

**Table 2:** Reclassification of patients based on the IL-6 concentration

Patients	Classification	
	Down	Up
Control	514 (57,4%)	381 (42,6%)
Severe course of CAP	41 (39,4%)	63 (60,6%)

The optimal cut-off concentrations that showed the highest prognostic accuracy (highest sensitivity and specificity) for IL-6, LBP and IL-10 in predicting a severe course of pneumonia were calculated using data from the ROC analyses. These data and the corresponding positive and negative predictive values are given in Table 3.

**Table 3:** Optimal cut-off values for cytokines, with sensitivity, specificity and positive and negative predictive values

	Cut-off value	Sensitivity	Specificity	PPV	NPV
Interleukin-6	27.2 pg/ml	70.5	60.4	0.057	0.984
Interleukin-10	3.15 pg/ml	67.3	58.1	0.051	0.981
LBP	22.45 μg/ml	55.2	66.6	0.053	0.978

Positive predictive values (PPV) and negative predictive values (NPV) were obtained using Bayes' formula, with a prevalence of 0.0326. LBP, lipopolysaccharide-binding protein.

### Microbiologic findings

The causative pathogen was identified in 321 (32.1%) of the patients. There was no significant difference (*P *= 0.537) in the rate of identification of causative pathogens in patients with antibiotic pretreatment (33.6%) compared with those without pretreatment (31.5%). The number of patients with known etiology was higher in severe courses of pneumonia (42.9% versus 30.8%, *P *= 0.013).

As shown in Tables 4 and 5, we identified 98 patients with typical bacterial infections, 155 patients with atypical bacterial infections, 23 patients with viral infections, and 45 patients with mixed infections. Mixed infections included combinations of two typical bacteria (seven patients), two atypical bacteria (five patients), typical and atypical bacteria (19 patients), typical bacteria and virus (10 patients), and atypical bacteria and viruses (four patients).

**Table 4:** Microbiologic findings for all patients

Microbial etiology	Number of patients		
	Inpatients	Outpatients	Total
Typical bacteria	65	33	98
*Streptococcus pneumonia*	39	17	56
*Haemophilus influenzae*	5	8	13
*Escherichia coli*	3	1	4
*Staphylococcus aureus*	5	3	8
*Moraxella catarrhalis*	1	1	2
*Enterobacter *spp.	2	1	3
*Pseudomonas aeruginosa*	6	0	6
*Citrobacter *spp.	0	1	1
*Klebsiella pneumoniae*	1	0	1
*Streptococcus pyogenes*	1	0	1
*Prevotella *spp.	1	0	1
*Acinetobacter *spp.	0	1	1
*Hafnia alvei*	1	0	1
Atypical pathogens	87	68	155
*Mycoplasma pneumoniae*	59	60	119
*Legionella *spp.	28	8	36
Viruses	9	14	23
Adenovirus	1	1	2
Influenza A	5	7	12
Influenza B	1	2	3
Respiratory syncyntial virus	1	3	4
Enterovirus	1	1	2
Mixed organisms	30	15	45
Unknown organism(s)	405	274	679

**Table 5:** Microbiologic findings for patients with severe pneumonia

Microbial etiology	Number of patients		
	Inpatients	Outpatients	Total
Typical bacteria	18	1	19
*Streptococcus pneumonia*	8	0	8
*Haemophilus influenza*	3	1	4
*Escherichia coli*	1	0	1
*Staphylococcus aureus*	2	0	2
*Enterobacter *spp.	1	0	1
*Pseudomonas aeruginosa*	2	0	2
*Streptococcus pyogenes*	1	0	1
Atypical pathogens	19	2	21
*Mycoplasma pneumonia*	10	2	12
*Legionella *spp.	9	0	9
Viruses	1	0	1
Influenza A	1	0	1
Mixed organisms	4	0	4
Unknown organism(s)	60	0	60

### Cytokine concentrations in patients with different infections

**Figure 4:**
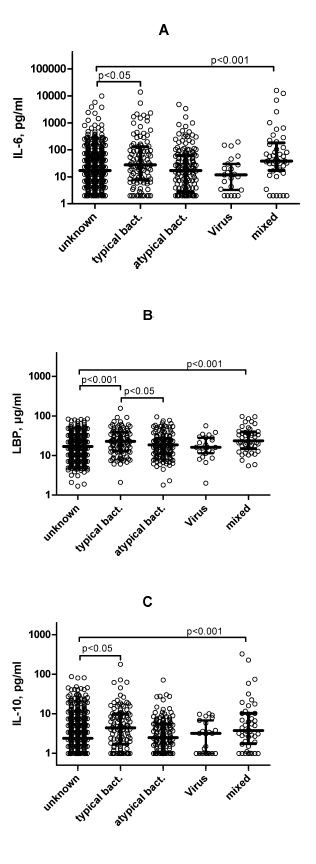
**Levels of cytokines on admission in patients with community-acquired pneumonia, and microbial etiology**. Microbial etiology was infection with typical bacteria, atypical bacteria, viruses or mixed organisms, or unknown etiology. The scatter plots represent all data. Median values with interquartile ranges are shown.

The highest cytokine concentrations were found in patients who were infected with typical bacteria such as *S. pneumoniae*. In Figure 4 the cytokine concentrations in patients with typical, atypical, viral and mixed infections are compared with those in patients infected with unknown pathogens.

We found significant differences in IL-6, IL-10 and LBP concentrations between patients with unknown pathogens and those with typical bacterial infections. The median values for IL-6 were 16.9 pg/ml versus 29.4 pg/ml (*P *= 0.042). The respective values for IL-10 were 2.4 pg/ml versus 4.2 pg/ml (*P *= 0.033), and for LBP they were 16.9 μg/ml versus 23.25 μg/ml (*P *< 0.001). In addition, significant differences in the LBP concentrations were found in patients with typical (23.25 μg/ml) and atypical bacterial infections (18.7 μg/ml, *P *< 0.012). We found 33 patients with Gram-negative and 65 patients with Gram-positive typical bacterial infections. Median LBP levels in patients with pneumonia caused by Gram-negative organisms were significantly lower (18.10 ± 11.76 μg/ml) compared with patients with pneumonia caused by Gram-positive bacteria (28.40 ± 25.38 μg/ml; *P *< 0.001). The IL-6 and IL-10 concentrations were similar in both patient groups.

## Discussion

This study demonstrated: (1) IL-6 and IL-10 concentrations at the time of patient admission reflected, similar to the CRB score, the severity of disease rather than prognosis, which is influenced not only by severity but also by age and co-morbidities of the patients; therefore, high IL-6 and/or IL-10 concentrations were found in patients with high CRB or CRB-65 scores; (2) the biomarkers used were inferior to CRB-65 score, but similar to the CRB score, for predicting a severe course of CAP; (3) the accuracy of both CRB and CRB-65 for predicting a severe course of pneumonia could be increased significantly by including the IL-6 measurements in a statistical model; (4) LBP had some predictive potential in etiology, but individual predictions could not be made.

Current CAP guidelines recommend that patients at increased risk of death should be hospitalized, and that ICU admission should be considered for patients with the highest risk. To date, several different pneumonia severity indexes, such as the pneumonia severity index, CURB, CURB-65, CRB and CRB-65, have been evaluated [16-18]. In addition, new biomarkers such as procalcitonin (PCT), proatrial natriuretic peptide (pro-ANP, copeptin), proatrial vasopressin (proAVP), proadrenomedullin (proADM) and proendothelin-1 (proET1) have been found to have similar, additive or superior predictive values for the risk assessment of CAP [19,20].

IL-6 and IL-10 play an important role in regulation of inflammatory reactions. Various studies have shown increased concentrations of these cytokines during pneumonia [21-24]. Elevated IL-6 and IL-10 levels have been directly associated with higher mortality, ICU admission and mechanical ventilation [25,26].

Increased LBP concentrations have been found in patients with severe sepsis caused by Gram-negative or Gram-positive bacterial infections or fungal infections [25,26]. Data concerning LBP concentration in CAP are scarce [27,28].

In contrast to most of the studies that have been published so far, we investigated the concentrations of IL-6, IL-10 and LBP in comparison with clinical and microbiologic findings in a large and carefully characterized study population, which included a large number of patients with a severe course of CAP; this was defined as mechanical ventilation, ICU treatment and/or death within 30 days of involvement.

In patients with a severe course of pneumonia, we found significantly increased IL-6 and LBP concentrations. In contrast to previous studies, which found new biomarkers such as PCT, pro-ANP, proAVP, proET1 and proADM to be good predictors of the course and the outcome of CAP, and which were similar or superior to CRB-65 score, we found a lower predictive potential for IL-6, IL-10 and LBP. As published recently, the predictive value of CRP was also lower than that of the CRB-65 score [10]. We were able to confirm these findings by calculating an AUC of 0.663 for differentiation of the combined end point and CRP (data not shown). The combination of CRB-65 score and IL-6 concentration increased the predictive value. This finding may be of limited value for the risk stratification of individual patients as there was a significant overlap in the distribution of IL-6 concentration in different patient groups [29]. Therefore, to test the value of IL-6 for the improving risk prediction we calculated the NRI for IL-6 in combination with CRB-65 score and compared it with CRB-65 score alone and found a significant net gain in reclassification by including IL-6.

On the other hand, we found a strong association between the IL-6 and IL-10 concentrations and high CRB scores (class 2-3). The operational characteristics of IL-6, IL-10 and CRB with respect to a severe course of CAP were similar to each other.

There are several factors that may reduce the prognostic potential of these parameters. First, the induction of IL-6 secretion precedes that of most other pro-inflammatory cytokines and is strongly controlled by anti-inflammatory cytokines such as IL-10. Second, all cytokines investigated in our study have a short plasma half-life of a few hours. Previous studies have shown that the mean levels of IL-6 and IL-10 decrease rapidly after clinical presentation in response to therapeutic interventions [30-32]. Thus, IL-6 and IL-10 are cytokines that characterize the acute clinical condition of patients. Therefore, and with respect to the high negative predictive values of 0.984, 0.981 and 0.978 for IL-6, IL-10 and LBP, respectively, normal cytokine concentrations might be used to identify patients who can be treated as outpatients.

Similar to our results, previous studies have shown that the CRB score fails to predict many severe courses of CAP. Consequently, it has been suggested that age ≥ 65 years should be included to identify patients at high risk because of potential comorbidities [25,26]; the resulting CRB-65 score has been shown to useful in characterizing the acute clinical condition and the risk of a severe course of CAP.

Cardiovascular markers such as copeptin (proAVP), proET1 or MR-proANP, or adrenomedullin (MR-proADM) which has multiple effects, reflect at least in part non-pulmonary comorbidities [19,20]. These biomarkers have the highest predictive value for death within 30 or 180 days. Inflammatory biomarkers such as PCT, which has a delayed concentration kinetic, have an intermediate predictive value that is comparable with the clinical CRB-65 score. We have shown that inflammatory markers that are rapidly regulated have a low predictive value that is comparable with that of the CRB score. Further studies should answer the questions: (1) does a combination these biomarkers with a cardiovascular marker further improve the prediction of the course of CAP, and (2) can these biomarkers be used to monitor disease progression? The latter question is not addressed within the CAPNETZ study because the timing of the biomarker measurements is not standardized, except that the blood samples were collected within 24 h after presentation at the study center. Under these conditions biomarkers that have short peak concentrations at defined disease states may have lower prognostic values compared with those with long-lasting elevated concentrations.

In CAP, the determination of the etiology of the microbial pathogen is essential to target therapeutic decisions. In our study population the etiology remained unknown in 67.9% of the patients; this is comparable with previous findings [11]. It has been suggested that diagnostic sensitivity is reduced by antibiotic pretreatment prior to hospital admission. For example, typical pathogens such as *S. pneumoniae *are easily missed after a single dose of antimicrobial treatment [11,33]. In our study we found no association between antibiotic pretreatment and identification of etiologic microbial pathogen. The identification rate was higher patients with a severe course of pneumonia.

The highest concentrations of LBP, IL-6 and IL-10 were found in patients infected with typical bacteria. In previous studies, elevated IL-6 concentrations were found in patients infected with *S. pneumoniae *[24,34]. The concentration of the anti-inflammatory IL-10 was increased in parallel to the increase in pro-inflammatory IL-6, and this reflects the amplification of antimicrobial as well as host-protective processes in CAP [35,36].

In our study, the LBP concentration was the best parameter to differentiate between typical and atypical or viral pathogens, and we found significantly higher LBP concentrations in patients with typical bacterial infections. In addition, in our study population, we found significantly higher LBP concentrations in patients with atypical infections compared with patients infected with an unknown pathogen. Masia *et al. *found LBP concentrations to be similar in patients infected with unknown pathogens, compared with those with viral and atypical infections [37]. Within the group of typical bacterial infections, we found higher LBP concentrations in patients infected with Gram-positive bacteria. This supports the previous finding that LBP is a non-specific marker of bacterial infection [25,26].

## Conclusions

In conclusion, we have shown that IL-6, LBP and IL-10 are biomarkers that are significantly increased in severe CAP. Increased IL-6 and IL-10 concentrations reflect an acute inflammation at time of admission and are associated with high CRB and CRB-65 scores. The predictive value for a severe course of CAP, defined by death, admission to ICU or mechanical ventilation, was lower than that of the CRB-65 score. However, the accuracy of both CRB and CRB-65 scores could be increased significantly by including the IL-6 measurements in the statistical model. IL-6 and IL-10 represent, with respect to CAP, a group of biomarkers that reflect the current clinical condition (that is, severity of disease) of patients; this is different from previously studied biomarkers, such as pro-ANP or proADM, which reflect cardiovascular comorbidities, and PCT, which might reflect persisting inflammatory reactions. LBP appeared to be the best parameter to predict a typical bacterial infection. Further studies should address the question of whether the predictive value can be improved by combined measurement of biomarkers from different groups, and whether these cytokines are useful biomarkers to monitor disease progression and response to therapeutic interventions.

## Abbreviations

AUC: Area under the curve; CAP: Community-acquired pneumonia; CFU: Colony-forming units; ICU: Intensive care unit; IL-6: Interleukin 6; IL-10: Interleukin 10; LBP: Lipopolysaccharide-binding protein; NRI: Net reclassification improvement; PCT: Procalcitonin; proADM: Proadrenomedullin; pro-ANP: Proatrial natriuretic peptide; proAVP: Proatrial vasopressin; proET1: Proendothelin-1; ROC: Receiver-operating characteristic.

## Competing interests

The authors declare that they have no competing interests.

## Authors' contributions

KZ and FB carried out the immunoassays, part of the statistical analyses and drafted the manuscript. PM performed part of the statistical analysis. TW and MP participated in its design and coordination. SE and MP participated in the design of the study and helped to draft the manuscript. All authors read and approved the final manuscript.

## Pre-publication history

The pre-publication history for this paper can be accessed here:

http://www.biomedcentral.com/1471-2466/12/6/prepub
